# Mediastinal Packing for Intractable Coagulopathy in Acute Aortic Dissection (Types 1 and 2 DeBakey): A Life-Saving Technique—Report of Experiences

**DOI:** 10.1155/2015/513617

**Published:** 2015-09-07

**Authors:** Aliasghar Moeinipour, Mehdi Fathi, Alireza Sepehri Shamloo, Shahram Amini, Hamid Hoseinikhah, Akram Kianinejad

**Affiliations:** ^1^Atherosclerosis Prevention Research Center, Mashhad University of Medical Sciences, Imam Reza Hospital, Iran; ^2^Imam Reza Hospital, Mashhad University of Medical Sciences, Iran; ^3^Cardiovascular ICU, Imam Reza Hospital, Mashhad University of Medical Sciences, Iran

## Abstract

Nonsurgical bleeding after complex thoracic aortic procedures (such as aortic dissection and aortic aneurysm) is a great challenge for cardiac surgeons because of severe coagulopathy, exsanguinous bleeding, and inevitable death. Temporary mediastinal packing (with sponge) in such cases is the only life-saving technique with good result in most cases. Herein, we presented three cases with acute aortic dissection with intractable bleeding that was successfully managed with mediastinal packing.

## 1. Introduction

Although aortic surgery for aortic dissection and aortic aneurysms has been advanced today, the major concern in aortic surgery is massive coagulopathy and intractable bleeding instead of any medical intervention like fresh frozen plasma, cryoprecipitate, and platelet [1, 2]. From the past, one of the last treatments for bleeding problem that was not controlled by routine options was temporary packing that can be applied in abdominal and pelvic surgery (especially in hepatic trauma). Rarely in cardiac surgery and especially aortic surgery, massive and intractable hemorrhage can occur which is resistant to all known treatments [1, 2]. In this complicated case, usually other drugs like protamine sulfate, tranexamic acid, and aprotinin have been used.

Systemic hypothermia and metabolic acidosis can aggregate hemorrhage especially in prolonged cardiac surgery like aortic surgery which usually have long time of cardiopulmonary bypass and total circulatory arrest [3, 4]. At the end of the procedure, after the surgeon has made sure that there is no surgical bleeding from suture lines, controlling of coagulopathy has started with blood products like fresh frozen plasma, platelets, cryoprecipitate, and protamine sulfate [5]. In cases that there is intractable and uncontrollable coagulopathy, there is an interesting life-saving option that includes temporary mediastinal packing with multiple sponges around the site of bleeding especially in aortic suture lines [6, 7] (Figure 3).

## 2. Cases Presentation

**Case 1 .**

A 35-year-old young man who was a known case of Marfan syndrome (familial) was admitted due to chest pain and echocardiography (TEE) ascending aortic dissection (Type 2 DeBakey) that extended from 1 cm of right coronary artery origin to distal thoracic aorta (Figure 2). Emergency Bentall procedure with use of composite graft was done but, at the end of surgery, massive bleeding and coagulopathy occurred despite all of the antihemorrhagic interventions (such as haemostatic powders and fibrin glue). Perforce for life saving of the patient, mediastinum (around ascending aorta) was packed with sponges for 24 hours and sternum was closed with 3 steel wires. After passing of this time, packing was removed and hemorrhage was completely controlled and finally the patient had a good result.

**Case 2 .**

We had faced acute aortic dissection (Type 2 DeBakey) in a 67-year-old man with prolonged history of hypertension and smoking. The patient suffered from severe interscapular chest pain where, in echocardiography (TEE), presence of ascending aortic dissection coexisting with severe aortic regurgitation was confirmed. Emergency Bentall procedure was done for him but due to long time of cardiopulmonary bypass and diseased tissue of aorta, uncontrollable bleeding has happened at the time of closure of sternum from all of the mediastinal and sternal structure. Severe coagulopathy was estimated in the etiology and after aggressive blood products were administrated, mediastinum (around ascending aorta) was packed with sponges and sternum was not closed and dressed in sterile drape. The patient was transferred to ICU and treatment of coagulopathy was continued for 24 hours. At the return to operating room, no active bleeding was seen. This patient was discharged from hospital at 10 days after surgery.

**Case 3 .**

A 56-year-old woman was referred with acute aortic dissection Type 1 DeBakey to our department (with symptoms of dyspnea and chest pain with past history of COPD). Aortic transverse arch replacement with implantation of carotid and left subclavian artery with total circulatory arrest was done for her and finally at the end of the procedure intractable coagulopathy was noticed despite multiple units of fresh frozen plasma and cryoprecipitate and platelets that were given to her (with local fibrin glue). After packing the sternum, few steel wires were inserted for temporary closure and the patient was then transferred to ICU. Packing was contained for 36 hours and in reevaluation of her in operating room hemorrhage was not seen. Patient had good recovery period and was discharged few days later.

## 3. Discussion

An aortic dissection is a serious condition in which the inner layer of the aorta tears. And blood surges through the tear, causing the inner and middle layers of the aorta to separate (dissect). If the blood-filled channel ruptures through the outside aortic wall, aortic dissection is often fatal.

Aortic dissection is uncommon. This condition most frequently occurs in men in their 60s and 70s. When an aortic dissection is detected early and treated promptly, the chance of survival greatly improves. Risk factors for aortic dissection include the following: (i) uncontrolled high blood pressure (hypertension), (ii) hardening of the arteries (atherosclerosis), (iii) weakened and bulging artery (preexisting aortic aneurysm), (iv) an aortic valve defect (bicuspid aortic valve), (v) narrowing of the aorta at birth (aortic coarctation), and (vi) Marfan syndrome, and rarely, aortic dissections are caused by blunt trauma such as during motor vehicle accidents [8].

A high index of suspicion is important in patients with predisposing risk factors, for example, hypertension, aneurismal disease of the aorta, or familial connective tissue disorders. In all patients, an ECG must be done to exclude acute myocardial infarction for which the treatment is very different and may involve thrombolytic therapy. Multiple modalities (CT, MRI scanning, and echocardiography) can be used to complement each other to facilitate diagnosis depending upon availability. Usually, type A dissections require surgery, while type B dissections are best managed medically [8]. A new management of acute aortic dissection is timely because of recent developments in diagnostic strategies (including biomarkers and imaging), endovascular graft design, and surgical treatment, which have led to a better understanding of the epidemiology, risk factors, and molecular nature of aortic dissection. Although open cardiac surgery is the ideal treatment for proximal acute aortic dissection, use of endovascular management is now established for complicated distal dissection and distal arch repair and has recently been discussed as a preemptive measure to avoid late complications by inducing aortic remodeling [8].

**Figure 1:**
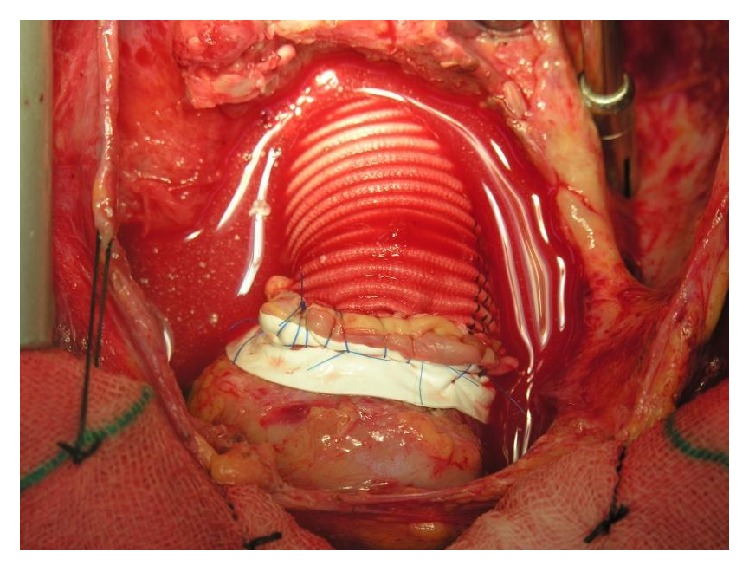
Bentall operation.

**Figure 2:**
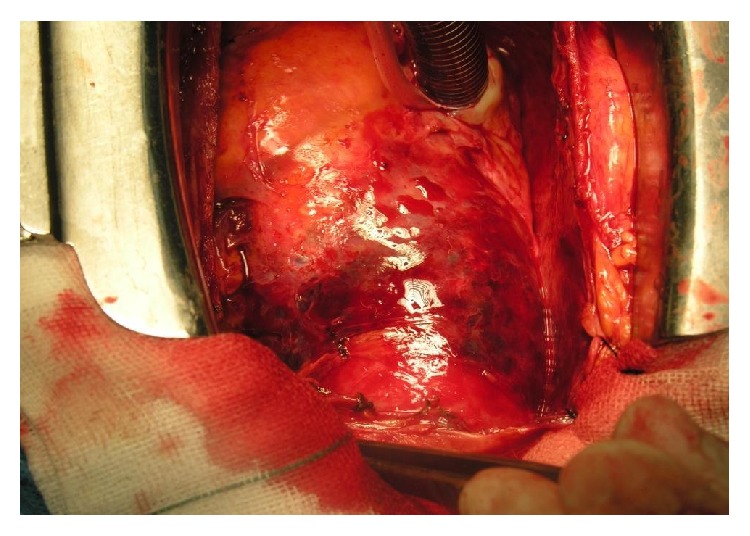
Ascending aortic dissection.

**Figure 3:**
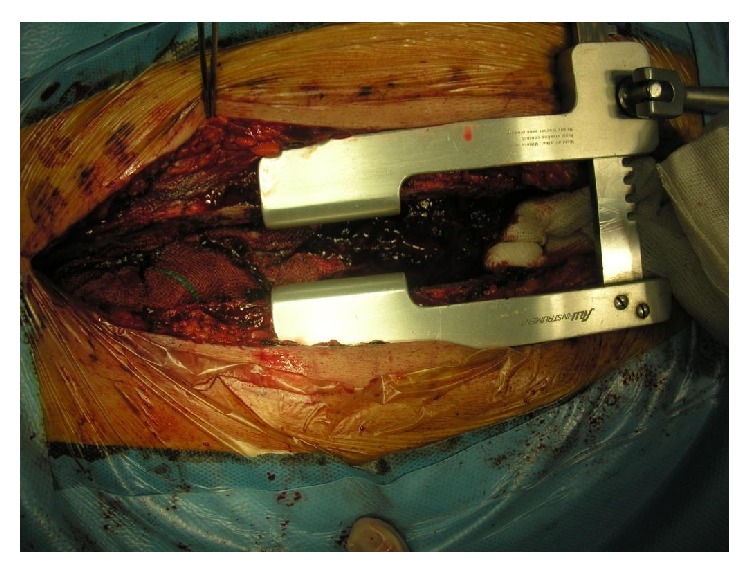
Mediastinal packing.

We have presented our technique in which at the end of procedure related to Aortic surgery like Bentall operation (Figure 1) and other complex aortic surgery when surgeon face to massive bleeding That is not possible for surgical control; potential etiology is coagulopathy and bleeding from deficit in coagulation system and needle orifice in aortic suture lines. In such cases, usually multiple units of fresh frozen plasma and platelets were given to patients but despite full correction of coagulopathy, intractable hemorrhage existed. Such condition can be lethal due to lack of blood product for replacing of ongoing blood loss and also coexisting acidosis and side effect of massive transfusion. Cardiac surgeon facing this challenging condition can apply temporary packing of mediastinal structure especially around aorta and also oozing sternal edge and pericardium and any site of diffuse bleeding. Sternum can be closed with few or multiple steel wires or sternum can be dressed with sterile drape. After transferring of patient to ICU, reverse of hypothermia is very important. Infusion of fresh frozen plasma and platelets and other blood products is required. This temporary packing is continued for about 12 to 36 hours but not longer. Usually at the return of patients to operating room, surgeons see that there is no active bleeding. The surgeon that has used this type of intervention for control of bleeding must notice that places of sponge pressure should not interfere with cardiac filling [9]. Then, sternum is temporarily closed with steel wire; in ICU care of patients, the important key is careful correction of hypothermia and metabolic acidosis; intensive care and hemodynamic monitoring of patients were essential with specific attention to signs of pericardial tamponade. Blood products usually are started at the arrival of patients to ICU [6, 10]. Usually, sternal packing was continued for 24 to 36 hours until complete hemostasis of patients, and after the surgeon suggests that correction of coagulopathy is completely done, patients return to operating room for reexploration [11]. After removal of sponges' gauze from mediastinum, massive irrigation with saline is done and later reevaluation of all of the suture lines and other possible bleeding points is done.

In a study by Bouboulis et al. in 1994, they evaluated 100 patients with temporary mediastinal packing for intractable bleeding. This study was done in 10 years' period. Hospital mortality was 8% and major complication and morbidity were sternal wound infection and sternal dehiscence and generalized sepsis [5]. They confirmed that mediastinal packing for cardiac procedure for massive bleeding that did not respond to earlier treatment could be a salvage option [5].

Mediastinal packing is a safe and life-saving option in management of intractable bleeding and coagulopathy after aortic surgery especially acute aortic dissection.
